# Limited Clinical Utility of Serum Prealbumin for Assessing DXA-Derived Lean Mass After Roux-en-Y Gastric Bypass: Longitudinal and Long-Term Cross-Sectional Analyses

**DOI:** 10.3390/nu18213580

**Published:** 2026-10-29

**Authors:** Sara Santini, Nathalie Vionnet, Elena Gonzalez-Rodriguez, Jérôme Pasquier, Styliani Mantziari, Lucie Favre

**Affiliations:** 1 Department of Endocrinology, Diabetology and Metabolism, Lausanne University Hospital, 1011 Lausanne, Switzerlandnathalie.vionnet@chuv.ch (N.V.); 2 Faculty of Biology and Medicine, University of Lausanne, Rue du Bugnon 21, 1011 Lausanne, Switzerland; elena.gonzalez-rodriguez@chuv.ch (E.G.-R.); styliani.mantziari@chuv.ch (S.M.); 3 Interdisciplinary Center for Bone Disease, Lausanne University Hospital, 1011 Lausanne, Switzerland; 4 NEXUS Personalized Health, ETH Zurich, 8092 Zurich, Switzerland; 5 Department of Visceral Surgery, Lausanne University Hospital, 1011 Lausanne, Switzerland

**Keywords:** Roux-en-Y gastric bypass, prealbumin, transthyretin, body composition, DXA, protein nutrition, metabolic-bariatric surgery

## Abstract

Background/Objectives: Despite recognized physiological limitations, serum prealbumin continues to be measured during nutritional follow-up after bariatric surgery, and low concentrations may be interpreted as indicating inadequate lean-mass preservation. We aimed to evaluate the clinical utility of serum prealbumin for identifying disproportionate DXA-derived lean mass loss during the first three years after Roux-en-Y gastric bypass (RYGB) and to examine its association with DXA-derived lean-mass measurements at long-term follow-up. Methods: In a longitudinal cohort of 178 adults, serum prealbumin and dual-energy X-ray absorptiometry (DXA)-derived body composition were assessed preoperatively, at 6 months, 1 year, and 3 years after surgery. Disproportionate lean mass loss was defined as lean mass accounting for >25% of total body weight lost. A complementary cross-sectional analysis examined associations between prealbumin and body composition in 310 participants assessed at a median of 8.8 years after RYGB. Results: Prealbumin decreased from an estimated 0.260 g/L preoperatively to 0.203 g/L at 6 months and partially recovered thereafter, while absolute lean mass continued to decline through 3 years. Prealbumin showed poor discrimination of disproportionate lean mass loss, with AUCs of 0.36, 0.28, and 0.48 at 6 months, 1 year, and 3 years, respectively, although estimates were imprecise because of the small number of participants meeting the criterion. The <0.20 g/L prealbumin threshold showed low sensitivity of 12–27%. Lower prealbumin concentrations were not significantly associated with the percentage of preoperative lean mass lost at any time point. In the long-term analysis, prealbumin was not meaningfully associated with DXA-derived lean mass measures after adjustment for sex, age, and time since surgery. Conclusions: Serum prealbumin showed limited discriminatory performance for identifying disproportionate DXA-derived lean mass loss during the first three years after RYGB, although estimates were imprecise because of the small number of events. In the long-term cross-sectional cohort, associations with DXA-derived lean-mass measurements were weak. These findings do not support the use of serum prealbumin as a surrogate marker of DXA-derived lean mass preservation after RYGB.

## 1. Introduction

Metabolic bariatric surgery (MBS) is currently the most effective treatment for severe obesity and is associated with substantial and sustained weight loss together with major improvements in obesity-related complications [1]. Alongside fat mass reduction, patients also experience a marked loss of lean mass (LM), particularly during the first postoperative year [2]. Although part of this loss is expected, excessive LM reduction may contribute to impaired physical function, reduced resting energy expenditure and potentially poorer long-term outcomes. Therefore, adequate nutritional monitoring after MBS is an essential component of lifelong follow-up [3]. However, diagnosing malnutrition in this population remains particularly challenging because intentional weight loss prevents the direct application of conventional diagnostic criteria such as the GLIM definition [4]. Clinicians often rely on surrogate biological markers despite the absence of robust evidence supporting their clinical relevance after MBS. Among these biomarkers, serum prealbumin remains one of the most frequently used markers in routine postoperative follow-up.

Prealbumin (transthyretin) is a hepatic transport protein that has traditionally been used as a biomarker of protein nutritional status because of its short half-life and responsiveness to nutritional and catabolic states. Although prealbumin is not an established marker of skeletal muscle mass, it has been proposed as a surrogate marker of lean mass, based on reported similarities between circulating prealbumin concentrations and age- and sex-related patterns of lean mass [5]. This proposed relationship, together with the continued use of prealbumin in postoperative nutritional monitoring, raises the clinically relevant question of whether circulating prealbumin provides information on lean-tissue preservation during substantial weight loss. However, prealbumin is a negative acute-phase reactant and its concentration is influenced by multiple factors unrelated to lean tissue, including inflammation, hepatic synthesis, metabolic state and recent nutritional intake. The recent ASPEN position paper concluded that visceral proteins should not be used in isolation as markers of nutritional status [6]. Despite these recognized limitations, serum prealbumin continues to be measured in postoperative bariatric care. Earlier clinical practice guidelines explicitly included prealbumin in routine biochemical monitoring after Roux-en-Y gastric bypass (RYGB) [7], and more recent publications continue to report its measurement as part of postoperative nutritional assessment [8,9]. A recent review also highlights that albumin and prealbumin remain commonly used to assess protein status after metabolic bariatric surgery, despite their limited reliability for this purpose [10]. This discrepancy between established physiological limitations and continued clinical use raises a practical question: whether serum prealbumin provides meaningful information on lean-mass preservation after bariatric surgery when assessed against an objective body-composition method such as DXA.

Several studies have described postoperative changes in serum prealbumin after MBS and most consistently reported a decline during the first postoperative year [11,12], followed by partial recovery thereafter [13,14]. More recently, two exploratory longitudinal studies involving 20 and 36 patients reported associations between lower serum prealbumin concentrations and lower bioimpedance-derived phase angle after bariatric surgery [15,16]. These observations raised the possibility that postoperative prealbumin concentrations might convey information related to nutritional or body-composition changes. However, phase angle does not directly quantify lean tissue, and these findings have not been validated in larger cohorts using reference methods for body composition assessment such as DXA.

We therefore aimed to evaluate the clinical utility of serum prealbumin as a surrogate marker of DXA-derived lean mass after RYGB, by assessing its ability to identify disproportionate lean-mass loss during the first three postoperative years and its association with lean-mass measures during long-term follow-up.

## 2. Materials and Methods

### 2.1. Study Design

This retrospective observational study was conducted at Lausanne University Hospital (CHUV), Switzerland, using data from the Cohort Obesity of Lausanne (COOL), an ongoing prospective cohort established in 2015 to investigate the long-term outcomes of MBS. The present analysis focused on participants who underwent Roux-en-Y gastric bypass (RYGB) and had eligible serum prealbumin and DXA-derived body-composition assessments available at one or more predefined study time points.

The primary analysis consisted of a longitudinal evaluation of the relationship between serum prealbumin and lean mass loss from before surgery to 6 months, 1 year, and 3 years after RYGB. This design enabled direct assessment of the relationship between changes in serum prealbumin and changes in lean mass over time. To evaluate the clinical relevance of serum prealbumin during long-term postoperative follow-up, a complementary cross-sectional analysis was performed using the latest available assessment obtained more than 3 years after RYGB. Participants could contribute to both analyses if they met the respective eligibility criteria; however, the longitudinal and cross-sectional analyses were based on distinct postoperative assessments.

### 2.2. Study Population

Patients who underwent primary laparoscopic RYGB at Lausanne University Hospital (CHUV) between May 2000 and June 2024 were eligible. All procedures were performed by the same experienced bariatric surgical team using a standardized surgical technique, thereby minimizing variability related to the operative procedure. All patients received standardized multidisciplinary perioperative care according to institutional practice and international guidelines. This included dietary counselling with a recommended protein intake of at least 60 g/day, routine vitamin and mineral supplementation, and structured recommendations regarding physical activity throughout follow-up.

Serum prealbumin and CRP were measured concomitantly from the same blood sample, allowing prealbumin concentrations to be interpreted in the context of the inflammatory status at the time of sampling. To minimize the potential influence of overt systemic inflammation on circulating prealbumin concentrations, assessments with CRP (or high-sensitivity CRP) >10 mg/L were excluded. Laboratory measurements were required to have been obtained within 3 months of the corresponding DXA-derived body-composition assessment. Assessments from patients with severe renal impairment (estimated glomerular filtration rate <30 mL/min/1.73 m^2^) were also excluded because of the potential influence of renal dysfunction on circulating serum prealbumin concentrations.

For the primary longitudinal analysis, participants were required to have preoperative serum prealbumin, CRP and DXA assessments together with at least one eligible postoperative evaluation performed at 6 months, 1 year, or 3 years after surgery.

For the complementary cross-sectional analysis, the latest eligible postoperative assessment performed more than 3 years after RYGB was retained for each participant. Participants were selected independently for the longitudinal and cross-sectional analyses according to the respective eligibility criteria. Consequently, 50 participants contributed to both analyses, but through distinct postoperative assessments; no DXA assessment was shared between the two analyses.

### 2.3. Measurements

Anthropometric measurements were obtained at the time of each DXA assessment. Body weight was measured with participants barefoot and wearing light clothing using a calibrated electronic scale (precision 0.1 kg), and height was measured using a wall-mounted stadiometer (precision 0.1 cm). Total weight loss (TWL) was calculated as the percentage reduction from preoperative body weight.

Blood samples were collected after an overnight fast and analyzed in the accredited clinical chemistry laboratory of Lausanne University Hospital. Serum prealbumin concentrations were measured by immunoturbidimetry (Cobas 8000, Roche Diagnostics, Rotkreuz, Switzerland). A prealbumin concentration <0.20 g/L, corresponding to the lower limit of the laboratory reference interval, was used as the clinical decision threshold, consistent with published literature [17]. C-reactive protein (CRP or hsCRP) and serum creatinine were measured as part of routine postoperative follow-up. Estimated glomerular filtration rate was calculated using the CKD-EPI equation.

Body composition was assessed by whole-body dual-energy X-ray absorptiometry (DXA) (Lunar iDXA, GE Healthcare, Madison, WI, USA) according to standardized procedures as previously described [18]. Total lean mass (LM) and fat mass (FM) were obtained from the manufacturer’s software. Appendicular lean mass index (ALMI) was calculated as appendicular lean mass divided by height squared (kg/m^2^), lean mass index (LMI) as total lean mass divided by height squared (kg/m^2^), and fat mass index (FMI) as total fat mass divided by height squared (kg/m^2^). Relative muscle mass was expressed as appendicular lean mass divided by body weight (ALM/weight).

To assess the contribution of lean mass loss to overall postoperative weight loss, the proportion of weight loss attributable to lean mass was calculated as:

$$\mathrm{LM}\;\mathrm{loss}\;\mathrm{as}\%\mathrm{weight}\;\mathrm{loss}=\frac{\mathrm{Preoperative}\;\mathrm{LM}\text{-}\mathrm{Follow}\;\mathrm{up}\;\mathrm{LM}}{\mathrm{Preoperative}\;\mathrm{body}\;\mathrm{weight}\text{-}\mathrm{Follow}\;\mathrm{up}\;\mathrm{body}\;\mathrm{weight}}\times 100$$

In addition, to quantify the magnitude of lean mass loss relative to the participant’s initial lean mass, the percentage of preoperative lean mass lost was calculated as:

$$\mathrm{Relative}\;\mathrm{LM}\;\mathrm{loss}\%=\frac{\mathrm{Preoperative}\;\mathrm{LM}\text{-}\mathrm{Follow}\;\mathrm{up}\;\mathrm{LM}}{\mathrm{Preoperative}\;\mathrm{LM}}\times 100$$

The first measure was used to identify disproportionate lean mass loss in the diagnostic-performance analysis, whereas the second expressed lean mass depletion irrespective of the amount of weight lost and was analyzed as a continuous outcome. Both were computed only in the longitudinal cohort, in which a preoperative assessment was available by design.

### 2.4. Statistical Analysis

Continuous variables are presented as mean (standard deviation [SD]), except for C-reactive protein (CRP) and time since surgery, which are presented as median [interquartile range] because of their skewed distributions. Categorical variables are presented as number (percentage). Descriptive results are reported overall and by sex, as appropriate.

Longitudinal trajectories were analyzed using separate linear mixed-effects models for each continuous anthropometric, biochemical, and body-composition outcome. Time was modeled as a categorical variable (preoperative, 6 months, 1 year, and 3 years), with fixed effects for time, sex, and their interaction, and a participant-specific random intercept to account for repeated measurements. Model-based marginal means at each time point were averaged over sex using the sex distribution of the preoperative cohort. Contrasts compared each postoperative assessment with the preoperative assessment; for total weight loss, for which a preoperative value is undefined, 6 months was used as the reference. Joint Wald chi-squared tests were used to assess time, sex, and the time-by-sex interaction. CRP and the binary indicator of prealbumin <0.20 g/L were summarized descriptively and were not modeled as longitudinal outcomes.

The ability of serum prealbumin to identify disproportionate lean mass loss was evaluated separately at 6 months, 1 year, and 3 years. Disproportionate loss was defined a priori as lean mass accounting for >25% of total body weight lost since surgery. This threshold was adopted as an operational, literature-informed definition for the present analysis rather than as a validated clinical diagnostic criterion. No consensus definition of excessive or disproportionate lean-mass loss after bariatric surgery is currently established. The threshold was selected because previous studies indicate that lean or fat-free mass typically accounts for approximately 20–25% of total weight loss after bariatric surgery [19]. A contribution exceeding 25% was therefore considered disproportionate for the purpose of the diagnostic-performance analysis. Receiver operating characteristic (ROC) curves used serum prealbumin measured at the corresponding assessment and were oriented a priori so that lower prealbumin indicated greater predicted risk. An area under the ROC curve (AUC) below 0.50 therefore indicates discrimination in the direction opposite to the prespecified hypothesis. Stratified bootstrap resampling with 10,000 replicates was used to obtain 95% confidence intervals (CIs) for the AUC. At the clinical threshold of prealbumin <0.20 g/L, sensitivity, specificity, positive predictive value, negative predictive value, and the positive likelihood ratio were calculated with corresponding 95% CIs. The maximum Youden index across all prealbumin thresholds was also calculated.

As a complementary analysis, lean mass lost since surgery was expressed as a percentage of preoperative lean mass and analyzed as a continuous outcome at each postoperative time point using linear regression adjusted for sex. Coefficients were expressed as the difference in percentage points of preoperative lean mass lost per 0.05 g/L lower serum prealbumin concentration. To assess the consequences of dichotomizing this outcome in the absence of an established clinical cut-off, sensitivity analyses calculated AUCs across thresholds ranging from 6% to 20% of preoperative lean mass lost. DeLong 95% CIs were used; thresholds yielding fewer than 15 cases were identified, and estimates were not displayed when either outcome group contained five or fewer participants.

Differences in preoperative characteristics according to the availability of an eligible 3-year assessment were explored by comparing participants with and without such an assessment. The latter group included both participants who had not yet reached the 3-year postoperative time point and those with missing assessments. Continuous variables were compared using Welch’s two-sample *t*-tests, CRP using the Wilcoxon rank-sum test, sex using a continuity-corrected two-sample test of proportions, and prealbumin <0.20 g/L using Fisher’s exact test. Standardized mean differences were additionally calculated for continuous variables.

Cross-sectional analyses used each participant’s most recent eligible assessment performed more than 3 years after surgery. Associations between serum prealbumin and total lean mass, lean mass as a percentage of body weight, ALMI, LMI, ALM/body weight, and FMI were assessed using ordinary least-squares regression. Model 1 was adjusted for sex, and Model 2 was additionally adjusted for age and time since surgery. Regression coefficients were expressed as the expected difference in each body-composition outcome per 0.05 g/L lower serum prealbumin concentration. Partial correlation coefficients adjusted for the same covariates were calculated with 95% CIs, and these intervals were examined to determine whether the 95% CIs excluded correlations of moderate or greater magnitude (|r| ≥ 0.30).

The association between serum prealbumin and ALMI was additionally examined according to time since surgery (3 to <5 years, 5 to <10 years, and ≥10 years), with adjustment for sex and age. Body-composition outcomes were also compared between participants with serum prealbumin <0.20 and ≥0.20 g/L using linear regression adjusted for sex, age, and time since surgery. Sex-specific scatterplots, ordinary least-squares regression lines, and unadjusted Pearson correlation coefficients were used for descriptive visualization of the associations between prealbumin and ALMI and between prealbumin and ALM/body weight.

Analyses used available observations for each outcome, with no imputation of missing values. Linear mixed-effects models used all available repeated measurements under a missing-at-random assumption, whereas other models used complete cases for the variables included in each analysis. All tests were two-sided, with *p* < 0.05 considered statistically significant. No adjustment for multiple testing was applied. Analyses were performed using R version 4.6.1 (R Foundation for Statistical Computing, Vienna, Austria), principally with the lme4, lmerTest, emmeans, car, and pROC packages.

## 3. Results

### 3.1. Longitudinal Cohort and Postoperative Changes

**Figure 1:**
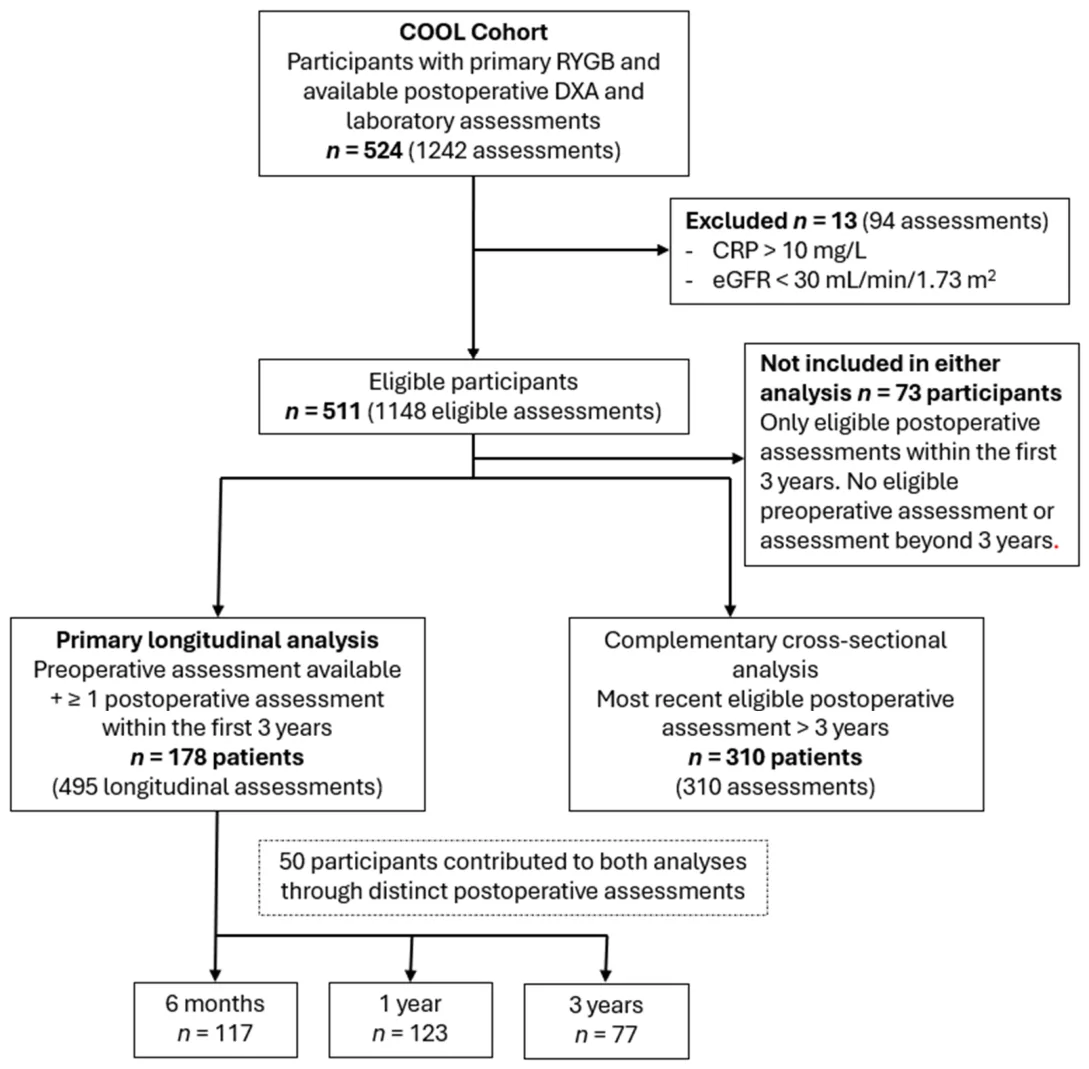
**Flow diagram of participant selection and analytical strategy.** The primary longitudinal analysis included participants with a preoperative assessment and at least one eligible postoperative assessment within the first 3 years after RYGB. The complementary cross-sectional analysis used the most recent eligible assessment performed more than 3 years after RYGB. Among the 511 participants with eligible assessments, 73 did not meet the inclusion criteria for either analysis because they had only postoperative assessments within the first three years after RYGB, without an eligible preoperative assessment or an assessment beyond three years. Fifty participants contributed to both analyses through distinct postoperative assessments; no DXA assessment was shared between the two analyses. Numbers in parentheses indicate the number of assessments with available DXA and corresponding laboratory measurements. For the cross-sectional analysis, only one assessment, the most recent eligible assessment performed more than 3 years after RYGB, was retained per participant. CRP, C-reactive protein; DXA, dual-energy X-ray absorptiometry; eGFR, estimated glomerular filtration rate; RYGB, Roux-en-Y gastric bypass.

Participant selection and the analytical strategy are summarized in Figure 1. The primary longitudinal cohort included 178 participants (124 women and 54 men) with a preoperative assessment and at least one eligible postoperative assessment within the first 3 years after RYGB. Preoperative characteristics are presented in Table 1. At 6 months, 1 year, and 3 years, eligible assessments were available for 117, 123, and 77 participants, respectively. The mean (SD) absolute interval between serum prealbumin measurement and the corresponding DXA assessment was 1.05 (0.81) months at 6 months, 1.98 (0.68) months at 1 year, and 1.30 (1.11) months at 3 years.

Serum prealbumin concentrations changed significantly over time (*p* < 0.001), decreasing from an estimated mean of 0.260 g/L preoperatively to 0.203 g/L at 6 months, followed by partial recovery to 0.223 g/L at 1 year and 0.226 g/L at 3 years (Table 2). The proportion of participants with prealbumin <0.20 g/L increased from 3% preoperatively to 44% at 6 months and was 22% at both 1 and 3 years. Median CRP concentrations were lower at all postoperative assessments than before surgery, including at 6 months when serum prealbumin concentrations reached their nadir.

Substantial changes in body composition occurred over the same period. Estimated total lean mass decreased by 5.5 kg (95% CI, 5.1–6.0) at 6 months, 5.9 kg (95% CI, 5.4–6.3) at 1 year, and 6.6 kg (95% CI, 6.0–7.1) at 3 years compared with the preoperative assessment, with a significant overall effect of time (*p* < 0.001). ALMI and LMI also decreased significantly over time (both *p* < 0.001). In contrast, lean mass expressed relative to body weight increased substantially after surgery, reaching an estimated increase of 14.0 percentage points (95% CI, 12.9–15.1) at 3 years, reflecting the proportionally greater loss of fat mass. Notably, the temporal patterns of serum prealbumin and lean mass differed: prealbumin reached its nadir at 6 months and subsequently partially recovered, whereas absolute lean mass and ALMI continued to decline through 3 years (Table 2).

Among the 178 participants in the longitudinal cohort, 77 (43%) had an eligible 3-year assessment. Of the 101 participants without an eligible 3-year assessment, 69 had not yet reached the 3-year postoperative time point at the time of data extraction, whereas 32 had missing assessments. Participants with and without a 3-year assessment had similar preoperative serum prealbumin concentrations and relative lean mass measures, although those with an available 3- year assessment had lower preoperative BMI and LMI (Supplementary Table S1).

### 3.2. Diagnostic Performance of Serum Prealbumin for Disproportionate Lean Mass Loss

**Figure 2:**
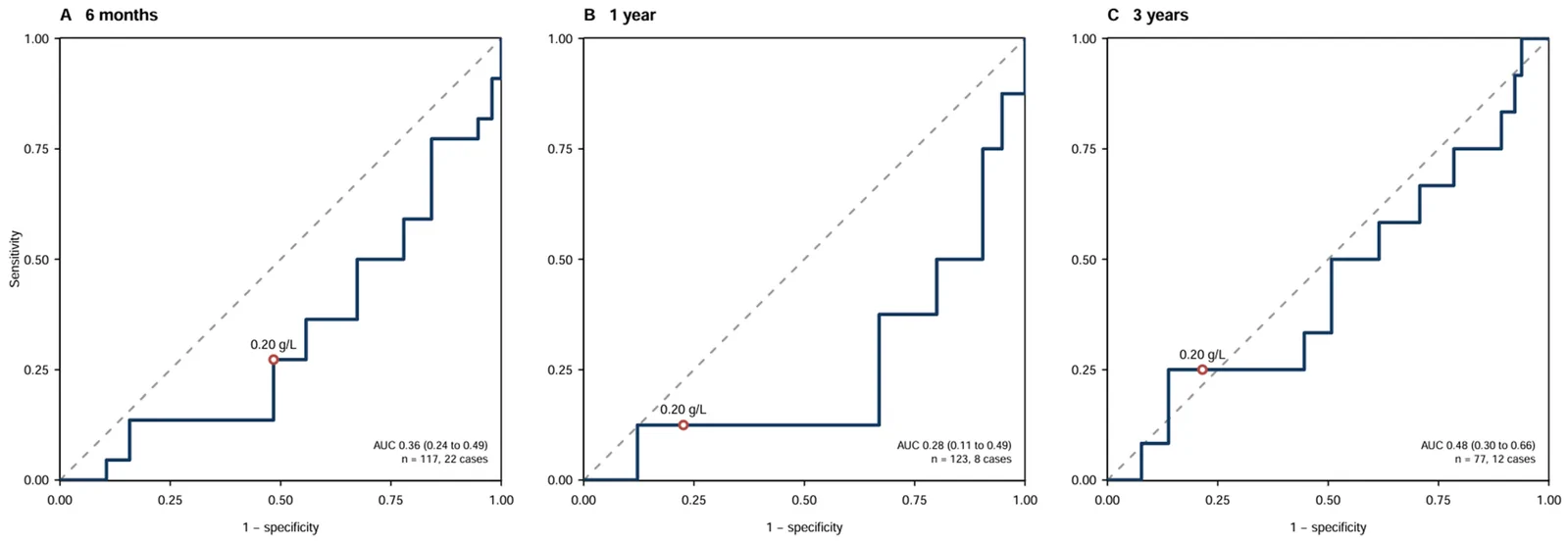
**Receiver operating characteristic curves for serum prealbumin to identify disproportionate lean mass loss at 6 months** (**A**)**, 1 year** (**B**)**, and 3 years** (**C**) **after Roux-en-Y gastric bypass.** Disproportionate lean mass loss was defined as lean mass accounting for >25% of total body weight lost since surgery. Prealbumin was oriented a priori so that lower concentrations represented greater predicted risk. Solid lines show the receiver operating characteristic curves, and the dashed diagonal line indicates no discrimination (AUC = 0.50). Open circles indicate the performance of the clinical prealbumin threshold of <0.20 g/L. Each panel reports the AUC with its 95% confidence interval, together with the number of participants assessed and the number meeting the disproportionate lean mass loss criterion. AUC, area under the receiver operating characteristic curve;.

Lean mass accounted for more than 25% of the weight lost since surgery in 22 of 117 participants (19%) at 6 months, 8 of 123 (7%) at 1 year, and 12 of 77 (16%) at 3 years (Table 3, Panel A). Serum prealbumin showed poor discrimination of disproportionate lean mass loss in the present sample, with AUCs of 0.36 (95% CI, 0.24–0.49) at 6 months, 0.28 (95% CI, 0.11–0.49) at 1 year, and 0.48 (95% CI, 0.30–0.66) at 3 years (Figure 2). However, the wide confidence intervals, particularly at 1 and 3 years, indicate substantial uncertainty in these estimates. The AUC estimates at 6 months and 1 year were below 0.50, with 95% confidence intervals also below this value, indicating discrimination in the direction opposite to the prespecified hypothesis. At 3 years, the confidence interval included 0.50. The small number of events, particularly at 1 and 3 years, limits the precision of these estimates.

At the clinical threshold of 0.20 g/L, sensitivity was 0.27 (95% CI, 0.13–0.48) at 6 months, 0.12 (95% CI, 0.02–0.47) at 1 year, and 0.25 (95% CI, 0.09–0.53) at 3 years, so that between three quarters and nine tenths of participants meeting the criterion were not identified. Specificity ranged from 0.52 to 0.78, positive predictive values from 0.04 to 0.18, and negative predictive values from 0.75 to 0.93. Positive likelihood ratios were 0.56 (95% CI, 0.28–1.15), 0.55 (95% CI, 0.09–3.57), and 1.16 (95% CI, 0.39–3.43), respectively. Even when the prealbumin threshold was optimized within the study sample, the maximum Youden index did not exceed 0.11, further indicating poor discriminatory performance.

When analyzed as a continuous outcome, lean mass lost since surgery represented 9.7 (4.8)%, 10.5 (4.7)%, and 12.0 (5.4)% of preoperative lean mass at 6 months, 1 year, and 3 years, respectively (Table 3, Panel B). Each 0.05 g/L lower prealbumin concentration was associated with an additional 0.51 percentage points (95% CI, −0.66 to 1.68) of preoperative lean mass lost at 6 months (*p* = 0.385), 0.79 percentage points (95% CI, −0.37 to 1.95) at 1 year (*p* = 0.179), and 0.92 percentage points (95% CI, −0.64 to 2.48) at 3 years (*p* = 0.242). All three estimates were in the direction expected if lower prealbumin reflected greater lean mass depletion, but none was statistically significant, and the magnitude was small: the upper limit of the most favorable confidence interval corresponds to 2.5 percentage points of preoperative lean mass, or approximately 1.2 kg in a participant with 50 kg of lean mass. In sensitivity analyses, the continuous outcome was dichotomized using thresholds ranging from 6% to 20% of preoperative lean mass lost. AUC estimates were generally close to 0.50 but approached 0.70 at some higher thresholds, particularly at 1 and 3 years (Supplementary Figure S1). However, these higher estimates were based on small numbers of events and had wide confidence intervals, limiting their interpretation.

### 3.3. Long-Term Cross-Sectional Associations Between Serum Prealbumin and Body Composition

The complementary cross-sectional analysis included 310 participants (242 women and 68 men) assessed more than 3 years after RYGB. Median time since surgery was 8.8 years [IQR, 5.5–13.6], with follow-up ranging from 3.0 to 24.1 years (Table 4). Mean serum prealbumin concentration was 0.225 (0.044) g/L, and 80 participants (26%) had concentrations <0.20 g/L.

After adjustment for sex, serum prealbumin was not significantly associated with any of the DXA-derived body-composition measures examined, including total lean mass, lean mass relative to body weight, ALMI, LMI, ALM/body weight, or FMI (Table 5, Panel A). Additional adjustment for age and time since surgery did not materially alter these findings. Partial correlations were small, and their 95% confidence intervals excluded correlations of moderate or greater magnitude (|r| ≥ 0.30) for all body-composition outcomes.

**Figure 3:**
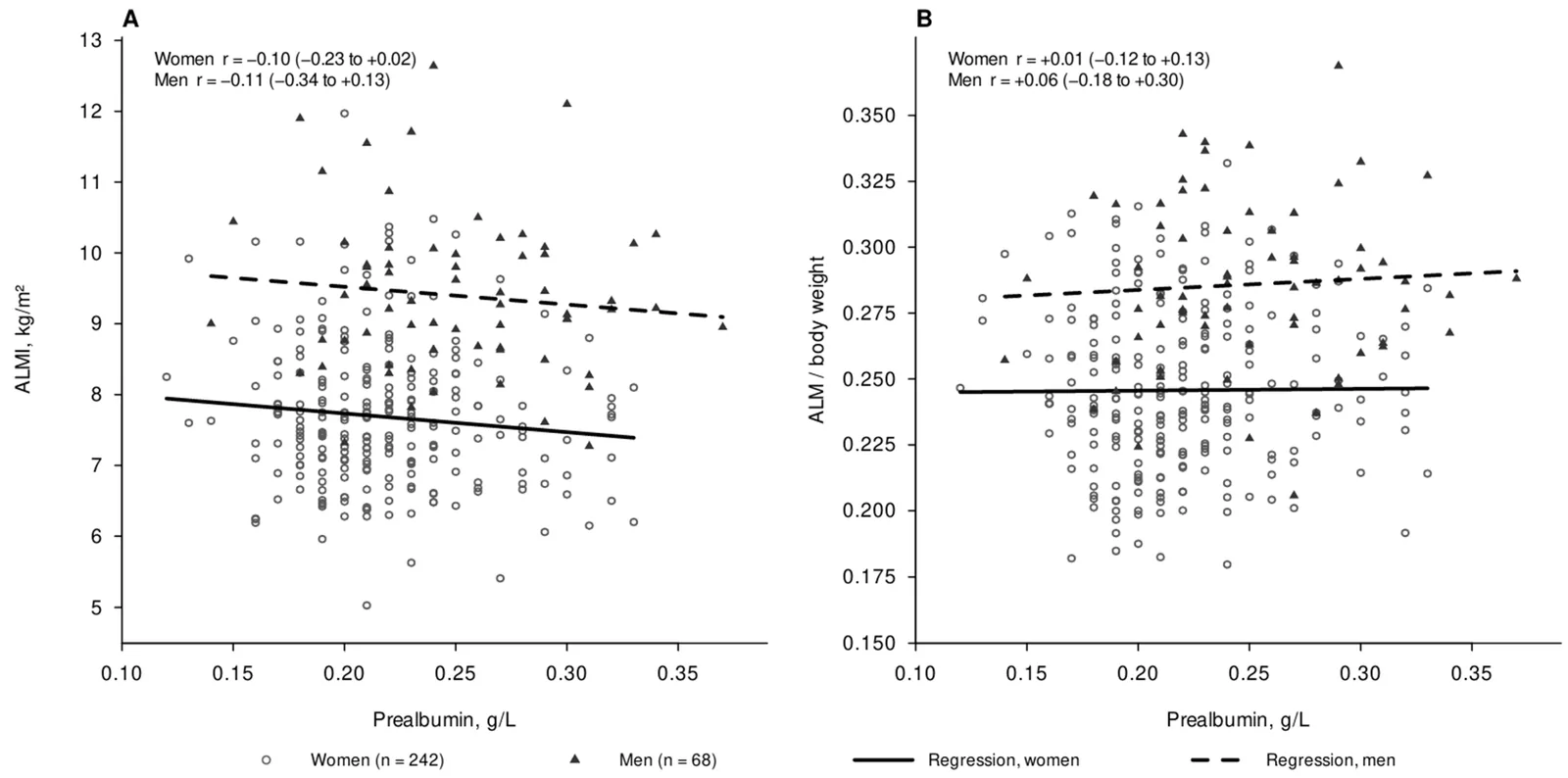
**Cross-sectional associations of serum prealbumin concentration with appendicular lean mass index** (**ALMI;** (**A**)) **and appendicular lean mass relative to body weight** (**B**)**, stratified by sex.** Points represent individual participants, and lines show sex-specific ordinary least-squares regression fits. Pearson correlation coefficients (r) with 95% confidence intervals are displayed for women and men separately. These analyses are descriptive and unadjusted; adjusted associations accounting for sex, age, and time since surgery are reported in Table 5. ALM, appendicular lean mass; ALMI, appendicular lean mass index; CI, confidence interval.

The association between serum prealbumin and ALMI remained non-significant when participants were stratified according to time since surgery (3 to <5 years, 5 to <10 years, and ≥10 years; Table 5, Panel B). Confidence intervals excluded a partial correlation of 0.30 in absolute value in the two later strata but not in the earliest one, which included only 59 participants. Sex-stratified descriptive analyses similarly showed no clear association between prealbumin and either ALMI or ALM/body weight (Figure 3).

Finally, participants with serum prealbumin <0.20 g/L did not have lower body-composition measures after adjustment for sex, age, and time since surgery (Table 5, Panel C). Unadjusted values suggested lower lean mass below the threshold, but this reflected the higher proportion of women in that group. For example, the adjusted difference in ALMI between participants with prealbumin <0.20 and ≥0.20 g/L was +0.08 kg/m^2^ (95% CI, −0.19 to 0.34; *p* = 0.572). No significant adjusted differences were observed for total lean mass, lean mass relative to body weight, LMI, ALM/body weight, or FMI.

## 4. Discussion

The present study evaluated whether serum prealbumin provides clinically meaningful information on lean mass preservation after RYGB. Three main findings emerged. First, serum prealbumin decreased markedly during the early postoperative period, reaching its nadir at 6 months and only partially recovering thereafter, whereas absolute lean mass continued to decline over the first 3 years. Second, prealbumin showed limited discriminatory performance for identifying participants with disproportionate lean mass loss, defined a priori as lean mass accounting for more than 25% of total weight lost, although the small number of participants meeting this criterion limits the precision of these estimates. This finding was consistent when lean mass loss was analyzed continuously as the percentage of preoperative lean mass lost, suggesting that the findings were not solely dependent on the selected dichotomous definition. In the long-term cross-sectional cohort, associations between serum prealbumin and DXA-derived body composition measures were weak: at a median of 8.8 years after RYGB, serum prealbumin was not associated with any of the DXA-derived body-composition measures examined. Importantly, these long-term analyses did not merely fail to detect statistically significant associations. The confidence intervals excluded correlations of moderate or greater magnitude for the main body-composition outcomes. Together, these findings indicate limited discriminatory performance of serum prealbumin for identifying disproportionate DXA-derived lean mass loss during the first three postoperative years and weak associations with DXA-derived lean mass measurements in the long-term cross-sectional cohort, with confidence intervals excluding correlations of moderate or greater magnitude.

The postoperative trajectory of prealbumin observed in our cohort is consistent with previous longitudinal bariatric studies. Andreu et al. followed 101 patients undergoing RYGB or sleeve gastrectomy for 12 months and reported an early decrease in prealbumin followed by partial recovery, with the proportion of patients with prealbumin <0.17 g/L decreasing from 39% at 4 months to 18% at 12 months [11]. Verger et al. prospectively followed 52 patients undergoing RYGB or sleeve gastrectomy for 1 year and similarly observed reduced prealbumin concentrations during postoperative follow-up [12]. Ritz et al., in 110 patients undergoing RYGB followed for 24 months, also reported a postoperative decrease in prealbumin with concentrations remaining below baseline at 2 years [13]. Previous studies therefore consistently show that low prealbumin concentrations are common after bariatric surgery and may persist beyond the period of most rapid weight loss. Importantly, these studies also questioned whether low postoperative prealbumin concentrations truly reflected protein malnutrition.

Given the known physiology of prealbumin, the absence of a strong relationship with lean mass may not appear unexpected. However, our findings extend previous evidence in several clinically relevant ways. Previous bariatric studies suggesting a relationship were small and relied on bioimpedance-derived phase angle [15,16]. In the present study, DXA-derived body-composition measurements obtained within predefined time windows and a diagnostic-performance framework allowed the clinical utility of prealbumin for identifying DXA-derived lean-mass loss to be evaluated. At the conventional threshold of 0.20 g/L, sensitivity was only 12–27%, meaning that most participants with disproportionate lean mass loss were not identified, while positive likelihood ratios close to 1 indicated little diagnostic information. The relatively high negative predictive values largely reflected the low prevalence of the target condition rather than strong discriminatory performance. Within the prespecified direction of lower prealbumin concentrations indicating greater risk of disproportionate lean-mass loss, no alternative prealbumin cut-off materially improved discrimination. Indeed, AUC estimates were numerically below 0.50 at 6 months and 1 year, indicating discrimination in the direction opposite to the prespecified hypothesis rather than an absence of discrimination. However, these findings should not be interpreted as establishing that higher prealbumin concentrations reliably predict greater lean-mass loss, particularly given the small number of events and wide confidence intervals. Finally, when lean mass loss was analyzed continuously as the percentage of preoperative lean mass lost, lower prealbumin was associated only with small and non-significant differences. Taken together, these complementary analyses suggest limited discriminatory utility of serum prealbumin for identifying disproportionate lean-mass loss after RYGB, while the small number of events and wide confidence intervals warrant caution in interpreting the diagnostic-performance estimates. This interpretation is further supported by the long-term cross-sectional analysis, in which associations between prealbumin and DXA-derived body-composition measures were small and their 95% confidence intervals excluded correlations of moderate or greater magnitude. The clinical relevance of these findings should be considered in the context of the continued use of prealbumin in postoperative bariatric care. Although its physiological limitations are well recognized, low prealbumin concentrations may still raise concerns about inadequate protein nutrition and lean-mass preservation. In our study, even after excluding overt systemic inflammation and severe renal impairment, low prealbumin concentrations did not reliably identify patients experiencing greater or disproportionate DXA-derived lean-mass loss. Conversely, normal concentrations do not exclude such loss. In the long-term cross-sectional cohort, low prealbumin concentrations were common but were not associated with lower DXA-derived lean mass measurements after adjustment for relevant covariates. These findings question the usefulness of routine prealbumin measurement when its intended purpose is to assess lean mass after RYGB. These findings also help place previous reports of an association between prealbumin and bioimpedance-derived parameters into perspective. Koehler et al. (n = 20) and Bortoli et al. (n = 36) reported positive associations between prealbumin and phase angle after bariatric surgery [15,16]. Phase angle is a raw bioelectrical parameter reflecting cellular integrity and fluid distribution rather than directly quantifying lean tissue. Importantly, phase angle, body composition and prealbumin may all change substantially during rapid postoperative weight loss, which may partly explain discrepancies between studies assessing different biological outcomes. An association between prealbumin and phase angle therefore does not necessarily imply that prealbumin reflects the amount of lean tissue lost.

Several mechanisms may explain why serum prealbumin and lean mass follow different postoperative trajectories. Prealbumin is a hepatic transport protein with a short half-life whose circulating concentration is influenced by factors beyond body protein stores, including inflammation, hepatic synthesis, energy and protein availability, and metabolic state [20,21]. Overt systemic inflammation is unlikely to explain the early postoperative decline observed in our study: assessments with CRP >10 mg/L were excluded, and median CRP concentrations were lower after surgery than before surgery, including at the 6-month prealbumin nadir. However, these observations do not exclude a contribution of residual low-grade inflammation. A similar pattern was reported by Andreu et al., in whom both hs-CRP and prealbumin decreased after bariatric surgery, while CRP remained inversely associated with prealbumin at 4 months [11]. Changes in energy and protein availability, together with broader hepatic and metabolic adaptations during rapid postoperative weight loss, may influence circulating prealbumin levels, although their specific contribution cannot be determined from our data. This uncertainty does not alter the observation that serum prealbumin did not provide clinically useful information on DXA-derived lean-mass loss. The early decrease in prealbumin may therefore partly reflect the profound negative energy balance and metabolic adaptation accompanying rapid postoperative weight loss rather than selective depletion of lean tissue. This interpretation is consistent with Ritz et al., who questioned whether the postoperative decrease in prealbumin represented protein malnutrition per se [13]. The temporal coincidence between the prealbumin nadir and the period of most rapid weight loss should therefore not be assumed to indicate that prealbumin reflects tissue depletion.

Lean mass loss after RYGB is itself a complex physiological phenomenon and cannot be equated with protein malnutrition. Published studies consistently document loss of lean tissue during the period of rapid postoperative weight reduction, although estimates vary according to the population and body-composition method used. Tamboli et al., in 29 patients assessed by DXA, reported that lean mass accounted for 27.8% of total weight loss at 1 year, with most lean mass loss occurring during the first 6 months [22]. Interestingly, this occurred despite a decrease rather than an increase in urinary 3-methylhistidine and estimated myofibrillar protein breakdown, while albumin concentrations remained normal. Davidson et al. similarly reported substantial early fat-free mass loss but found that skeletal muscle mass was largely maintained between 1 and 5 years after bariatric surgery [23]. Importantly, lean mass or fat-free mass and skeletal muscle are related but distinct body-composition compartments and changes in lean mass should not be interpreted as equivalent to changes in skeletal muscle. Furthermore, measures of tissue quantity alone do not capture muscle quality, strength or physical performance [24]. These distinctions are particularly important in the context of substantial weight loss, where changes in DXA-derived lean mass may reflect several lean-tissue compartments and do not, by themselves, establish protein malnutrition or sarcopenia. The proportion of weight loss attributable to lean mass therefore provides a pragmatic measure of the composition of weight loss, while recognizing that the >25% criterion used in the present study is an operational, literature-informed definition rather than a validated diagnostic threshold for excessive lean-mass loss.

The long-term findings further strengthen this interpretation. At a median of 8.8 years after RYGB, approximately one quarter of participants still had serum prealbumin concentrations below 0.20 g/L. This is consistent with previous evidence that low concentrations may persist well beyond the active weight-loss phase. Voglino et al. reported prealbumin <0.20 g/L in 23.1% of patients 3 years after RYGB, whereas hypoalbuminemia was considerably less frequent [14]. Yet in our cohort, participants with prealbumin <0.20 g/L did not have lower values for any of the DXA-derived body-composition measures examined. After adjustment for sex, age, and time since surgery, associations remained small, and the confidence intervals of the main analyses excluded correlations of moderate or greater magnitude. Thus, a low prealbumin concentration measured several years after RYGB should not, in isolation, be interpreted as evidence of lower DXA-derived lean mass. Conversely, a normal prealbumin concentration cannot provide reassurance regarding lean-mass preservation, since most participants meeting the disproportionate lean-mass-loss criteria were not identified by the conventional threshold.

These findings do not support the use of serum prealbumin as a surrogate marker of DXA-derived lean mass after RYGB. Importantly, our study was not designed to evaluate the value of prealbumin for other aspects of postoperative nutritional assessment, and our findings should not be extrapolated to these contexts. Similarly, DXA-derived lean mass does not capture skeletal muscle quality or function, which were not assessed in the present study.

Our findings are consistent with the broader evolution of nutritional assessment, in which circulating visceral proteins are increasingly distinguished from direct assessment of nutritional phenotype. Contemporary recommendations no longer support albumin or prealbumin as isolated markers of nutritional status and instead emphasize a multidimensional nutritional assessment incorporating clinical and functional measures [6]. The 2025 ESPEN guideline on clinical nutrition in surgery explicitly states that visceral proteins should no longer be considered markers of nutritional status and emphasizes direct assessment of the muscle compartment when clinically appropriate [25]. Similarly, the updated GLIM framework includes reduced muscle mass among the phenotypic criteria for malnutrition [26]. This issue is particularly relevant after bariatric surgery, where intentional weight loss complicates interpretation of conventional malnutrition criteria and increases the importance of assessing body composition and muscle status in their appropriate clinical context. Recent bariatric literature further suggests that albumin and prealbumin are insufficiently sensitive to the adequacy of protein intake and should not be used to guide protein recommendations [10]. Taken together with our findings, these recommendations support an approach in which postoperative nutritional assessment integrates dietary intake, clinical status, body composition, and muscle function rather than interpreting serum prealbumin as a surrogate of lean-mass preservation.

### Strengths and Limitations

The present study has several strengths. It includes a relatively large and well-characterized RYGB population with biochemical and DXA assessments obtained within predefined time windows, a longitudinal analysis spanning the period of greatest postoperative weight loss and a complementary cross-sectional analysis extending to more than two decades after surgery. Potential confounding by overt systemic inflammation and severe renal dysfunction was minimized through predefined exclusion criteria and sex was systematically accounted for because both prealbumin concentrations and lean-mass measures differ substantially between women and men. Importantly, we evaluated diagnostic performance rather than relying solely on statistical correlations and examined lean mass loss both using a literature-informed dichotomous criterion and as a continuous outcome, reducing dependence on any single definition of disproportionate lean mass loss.

Several limitations should be acknowledged. First, the observational, single-center design precludes determination of the mechanisms underlying postoperative changes in prealbumin and may limit generalizability. Individual protein and energy intake, physical activity and detailed dietary data were not available, limiting our ability to investigate the mechanisms underlying changes in serum prealbumin. As with any observational study, the possibility of residual confounding due to unmeasured or incompletely measured postoperative factors cannot be excluded. Second, although serum prealbumin and CRP were measured concomitantly, blood sampling and DXA were not necessarily performed on the same day. For postoperative assessments, an interval of up to three months was permitted, potentially introducing temporal misclassification, particularly during rapid early postoperative weight loss. However, the actual mean absolute interval at the 6-month assessment was approximately one month. Third, DXA quantifies lean soft tissue rather than skeletal muscle specifically and does not assess muscle composition, quality, strength or physical performance. Accordingly, our findings cannot be extrapolated to these dimensions of muscle health or to the diagnosis of sarcopenia. Fourth, an eligible 3-year assessment was available for 77 of the 178 participants in the longitudinal cohort. Among those without a 3-year assessment, 69 had not yet reached this postoperative time point, while 32 had missing assessments. Thus, incomplete 3-year data largely reflected the duration of available follow-up rather than attrition. Nevertheless, participants with and without an eligible 3-year assessment differed in baseline BMI and LMI, and potential selection bias cannot be excluded. These differences may limit the representativeness of the 3-year estimates. Fifth, the number of participants meeting the prespecified disproportionate lean-mass loss criterion was small, particularly at 1 year (n = 8) and 3 years (n = 12), resulting in limited precision and wide confidence intervals for the corresponding AUC and sensitivity estimates. These diagnostic-performance estimates should therefore be interpreted cautiously. Furthermore, nonsignificant associations should not be interpreted as evidence of equivalence or complete absence of association.

## 5. Conclusions

Serum prealbumin showed limited discriminatory performance for identifying disproportionate DXA-derived lean-mass loss during the first three years after RYGB, although the small number of events limits the precision of these estimates. In the long-term cross-sectional cohort, associations between serum prealbumin and DXA-derived lean-mass measurements were weak, with confidence intervals excluding correlations of moderate or greater magnitude. These findings do not support the use of serum prealbumin as a surrogate marker of DXA-derived leanmass after RYGB. Future studies should focus on identifying clinically applicable approaches that can better characterize musculoskeletal adaptation to weight loss, integrating changes in muscle mass with muscle quality and physical function.

## Figures and Tables

**Table 1:** **Preoperative characteristics of the longitudinal cohort**.

	Women	Men	All
***n***	**124**	**54**	**178**
Age, years	43.6 (11.5)	43.1 (10.0)	43.4 (11.0)
Height, m	1.62 (0.06)	1.77 (0.06)	1.67 (0.09)
Body weight, kg	110.6 (16.4)	139.8 (25.7)	119.4 (23.8)
BMI, kg/m^2^	41.9 (5.3)	44.6 (6.9)	42.7 (5.9)
**Biochemistry**			
Prealbumin, g/L	0.255 (0.040)	0.269 (0.039)	0.260 (0.040)
Prealbumin < 0.20 g/L, *n* (%)	5 (4)	0 (0)	5 (3)
CRP, mg/L	4.3 [2.8–6.8]	3.3 [1.8–5.0]	4.0 [2.3–6.4]
**Body composition** (**DXA**)			
Total lean mass, kg	50.0 (5.8)	71.2 (8.3)	56.4 (11.8)
Lean mass, % of body weight	45.9 (4.1)	52.1 (5.6)	47.8 (5.4)
Total fat mass, kg	57.2 (12.0)	63.8 (18.6)	59.2 (14.6)
Fat mass, % of body weight	51.7 (4.3)	45.3 (6.0)	49.8 (5.7)
ALMI, kg/m^2^	9.12 (0.96)	11.20 (1.08)	9.75 (1.39)
LMI, kg/m^2^	18.96 (1.74)	22.75 (2.00)	20.11 (2.52)
FMI, kg/m^2^	21.69 (4.19)	20.32 (5.38)	21.27 (4.61)
ALM/body weight	0.219 (0.020)	0.255 (0.030)	0.230 (0.029)

Data are presented as mean (SD), median [interquartile range], or n (%), as appropriate. ALM, appendicular lean mass; ALMI, appendicular lean mass index; BMI, body mass index; CRP, C-reactive protein; DXA, dual-energy X-ray absorptiometry; FMI, fat mass index; LMI, lean mass index; SD, standard deviation.

**Table 2:** **Anthropometric, biochemical, and body-composition changes over the first three years after Roux-en-Y gastric bypass in the longitudinal cohort**.

	Assessment						***p*** **Value**
	Preoperative	6 Months	1 Year	3 Years	**Time**	**Sex**	**Sex × Time**
**Observations, *n***	**178**	**117**	**123**	**77**	—	—	—
**Anthropometry**							
**Body weight, kg**	119.4 (23.8)	89.2 (19.5)	79.9 (16.3)	78.8 (15.5)	<0.001	<0.001	<0.001
Estimated mean (SE)	119.4 (1.3)	87.7 (1.4)	81.0 (1.4)	81.5 (1.5)			
Δ vs. preoperative (95% CI)	reference	−31.7 (−33.6, −29.9)	−38.4 (−40.3, −36.6)	−38.0 (−40.2, −35.7)			
**BMI, kg/m^2^**	42.7 (5.9)	31.9 (5.1)	28.5 (4.5)	28.2 (4.4)	<0.001	0.033	0.472
Estimated mean (SE)	42.7 (0.4)	31.4 (0.4)	29.0 (0.4)	29.1 (0.5)			
Δ vs. preoperative (95% CI)	reference	−11.4 (−12.0, −10.7)	−13.7 (−14.3, −13.1)	−13.6 (−14.4, −12.8)			
**Total weight loss, %**	—	26.2 (7.2)	31.7 (8.2)	31.7 (10.0)	<0.001	0.719	0.512
Estimated mean (SE)	—	26.7 (0.7)	31.9 (0.7)	31.4 (0.8)			
Δ vs. 6 months (95% CI)	—	reference	+5.2 (+3.8, +6.6)	+4.7 (+3.2, +6.3)			
**Biochemistry**							
**Prealbumin, g/L**	0.260 (0.040)	0.202 (0.038)	0.224 (0.037)	0.231 (0.040)	<0.001	0.032	0.582
Estimated mean (SE)	0.260 (0.003)	0.203 (0.003)	0.223 (0.003)	0.226 (0.004)			
Δ vs. preoperative (95% CI)	reference	−0.056 (−0.063, −0.050)	−0.037 (−0.043, −0.031)	−0.033 (−0.041, −0.026)			
**Prealbumin < 0.20 g/L, *n*** (**%**)	5 (3)	52 (44)	27 (22)	17 (22)	—	—	—
**CRP, mg/L**	4.0 [2.3–6.4]	1.0 [0.6–1.6]	0.6 [0.5–1.0]	0.6 [0.6–1.2]	—	—	—
**Body composition** (**DXA**)							
**Total lean mass, kg**	56.4 (11.8)	51.7 (11.0)	50.2 (10.8)	48.2 (9.7)	<0.001	<0.001	0.008
Estimated mean (SE)	56.4 (0.5)	50.9 (0.5)	50.6 (0.5)	49.9 (0.5)			
Δ vs. preoperative (95% CI)	reference	−5.5 (−6.0, −5.1)	−5.9 (−6.3, −5.4)	−6.6 (−7.1, −6.0)			
**Lean mass, % of body weight**	47.8 (5.4)	58.6 (7.7)	63.4 (8.3)	61.6 (7.4)	<0.001	<0.001	0.015
Estimated mean (SE)	47.8 (0.5)	58.6 (0.5)	63.3 (0.5)	61.8 (0.6)			
Δ vs. preoperative (95% CI)	reference	+10.9 (+10.0, +11.8)	+15.5 (+14.6, +16.4)	+14.0 (+12.9, +15.1)			
**Total fat mass, kg**	59.2 (14.6)	34.5 (12.1)	26.8 (10.1)	28.1 (9.7)	<0.001	<0.001	<0.001
Estimated mean (SE)	59.2 (0.9)	33.9 (1.0)	27.4 (1.0)	29.1 (1.1)			
Δ vs. preoperative (95% CI)	reference	−25.4 (−27.0, −23.8)	−31.8 (−33.4, −30.3)	−30.2 (−32.0, −28.3)			
**Fat mass, % of body weight**	49.8 (5.7)	38.2 (8.1)	33.2 (8.7)	35.1 (7.8)	<0.001	<0.001	0.017
Estimated mean (SE)	49.8 (0.5)	38.1 (0.5)	33.3 (0.5)	35.0 (0.6)			
Δ vs. preoperative (95% CI)	reference	−11.7 (−12.6, −10.7)	−16.5 (−17.5, −15.6)	−14.8 (−15.9, −13.6)			
**ALMI, kg/m^2^**	9.75 (1.39)	8.47 (1.28)	8.08 (1.27)	7.80 (1.17)	<0.001	<0.001	0.184
Estimated mean (SE)	9.75 (0.07)	8.36 (0.08)	8.15 (0.08)	8.04 (0.09)			
Δ vs. preoperative (95% CI)	reference	−1.39 (−1.50, −1.28)	−1.59 (−1.70, −1.49)	−1.70 (−1.84, −1.57)			
**LMI, kg/m^2^**	20.11 (2.52)	18.42 (2.40)	17.82 (2.26)	17.19 (2.01)	<0.001	<0.001	0.890
Estimated mean (SE)	20.11 (0.13)	18.12 (0.14)	18.00 (0.14)	17.74 (0.15)			
Δ vs. preoperative (95% CI)	reference	−1.98 (−2.15, −1.82)	−2.11 (−2.27, −1.94)	−2.37 (−2.56, −2.17)			
**FMI, kg/m^2^**	21.27 (4.61)	12.39 (4.06)	9.68 (3.64)	10.16 (3.52)	<0.001	0.074	0.679
Estimated mean (SE)	21.27 (0.31)	12.17 (0.34)	9.94 (0.34)	10.48 (0.38)			
Δ vs. preoperative (95% CI)	reference	−9.11 (−9.66, −8.55)	−11.33 (−11.88, −10.79)	−10.80 (−11.45, −10.14)			
**ALM/body weight**	0.230 (0.029)	0.268 (0.033)	0.285 (0.038)	0.279 (0.036)	<0.001	<0.001	0.001
Estimated mean (SE)	0.230 (0.002)	0.268 (0.002)	0.285 (0.002)	0.280 (0.003)			
Δ vs. preoperative (95% CI)	reference	+0.039 (+0.035, +0.042)	+0.055 (+0.051, +0.059)	+0.050 (+0.046, +0.054)			

Observed data are presented as mean (SD), except for CRP, presented as median [interquartile range], and prealbumin <0.20 g/L, presented as n (%). Estimated means (SE) and contrasts were derived from linear mixed-effects models including time, sex, a sex-by-time interaction, and a participant-specific random intercept. Contrasts are relative to the preoperative assessment, except for total weight loss, for which 6 months was the reference. ALM, appendicular lean mass; ALMI, appendicular lean mass index; BMI, body mass index; CI, confidence interval; CRP, C-reactive protein; DXA, dual-energy X-ray absorptiometry; FMI, fat mass index; LMI, lean mass index; SD, standard deviation; SE, standard error.

**Table 3:** **Diagnostic performance of serum prealbumin for disproportionate lean mass loss after Roux-en-Y gastric bypass**.

	6 Months	1 Year	**3 Years**
**Panel A. Detection of excessive lean mass loss, defined as more than 25% of the weight lost**			
**Patients assessed, *n***	**117**	**123**	**77**
**Patients meeting the criterion, *n*** (**%**)	**22** (**19**)	**8** (**7**)	**12** (**16**)
**AUC of prealbumin** (**95% CI**)	**0.36** (**0.24 to 0.49**)	**0.28** (**0.11 to 0.49**)	**0.48** (**0.30 to 0.66**)
Prealbumin < 0.20 g/L			
True positives/false positives	6/46	1/26	3/14
False negatives/true negatives	16/49	7/89	9/51
Sensitivity (95% CI)	0.27 (0.13 to 0.48)	0.12 (0.02 to 0.47)	0.25 (0.09 to 0.53)
Specificity (95% CI)	0.52 (0.42 to 0.61)	0.77 (0.69 to 0.84)	0.78 (0.67 to 0.87)
Positive predictive value (95% CI)	0.12 (0.05 to 0.23)	0.04 (0.01 to 0.18)	0.18 (0.06 to 0.41)
Negative predictive value (95% CI)	0.75 (0.64 to 0.84)	0.93 (0.86 to 0.96)	0.85 (0.74 to 0.92)
Positive likelihood ratio (95% CI)	0.56 (0.28 to 1.15)	0.55 (0.09 to 3.57)	1.16 (0.39 to 3.43)
Maximum Youden index across all cut-offs	0.00	0.00	0.11
**Panel B. Association with the percentage of preoperative lean mass lost, analysed as a continuous outcome**			
**Patients assessed, *n***	**117**	**123**	**77**
**Lean mass lost, % of preoperative value**	**9.7** (**4.8**)	**10.5** (**4.7**)	**12.0** (**5.4**)
**Difference per 0.05 g/L lower prealbumin,**	**+0.51** (**−0.66 to +1.68**)	**+0.79** (**−0.37 to +1.95**)	**+0.92** (**−0.64 to +2.48**)
percentage points (95% CI)			
*p* value	0.385	0.179	0.242

Panel A evaluates the ability of serum prealbumin to identify disproportionate lean mass loss, defined as lean mass accounting for >25% of total body weight lost since surgery. Prealbumin was analyzed both as a continuous variable using the area under the receiver operating characteristic curve and at the clinical threshold of <0.20 g/L. Panel B examines the percentage of preoperative lean mass lost as a continuous outcome; regression coefficients are adjusted for sex and expressed as the additional percentage points of preoperative lean mass lost per 0.05 g/L lower serum prealbumin concentration. Data for lean mass lost are presented as mean (SD). AUC, area under the receiver operating characteristic curve; CI, confidence interval; SD, standard deviation.

**Table 4:** **Characteristics of the cross-sectional cohort assessed more than 3 years after Roux-en-Y gastric bypass**.

	Women	Men	All
*n*	242	68	310
**Demographics and anthropometry**			
Age, years	51.2 (11.0)	52.8 (9.2)	51.6 (10.6)
Height, m	1.61 (0.07)	1.75 (0.07)	1.64 (0.09)
Preoperative body weight, kg	115.5 (18.8)	139.7 (22.4)	120.8 (22.0)
Body weight at assessment, kg	83.9 (17.7)	104.5 (21.1)	88.4 (20.4)
BMI at assessment, kg/m^2^	31.8 (6.2)	33.3 (5.8)	32.1 (6.1)
Total weight loss, %	27.2 (10.9)	25.0 (9.9)	26.7 (10.8)
**Time since surgery**			
Median, years [IQR]	8.8 [5.5–13.6]	8.9 [5.4–13.4]	8.8 [5.5–13.6]
Range, years	3.0–24.1	3.0–22.5	3.0–24.1
3 to <5 years, *n* (%)	44 (18)	15 (22)	59 (19)
5 to <10 years, *n* (%)	96 (40)	23 (34)	119 (38)
≥10 years, *n* (%)	102 (42)	30 (44)	132 (43)
**Biochemistry**			
Prealbumin, g/L	0.218 (0.040)	0.250 (0.048)	0.225 (0.044)
Prealbumin < 0.20 g/L, *n* (%)	73 (30)	7 (10)	80 (26)
CRP, mg/L (*n* = 309)	0.8 [0.6–2.1]	1.0 [0.6–1.4]	0.8 [0.6–2.0]
eGFR, mL/min/1.73 m^2^ (*n* = 305)	86 (9)	81 (12)	85 (10)
**Body composition** (**DXA**)			
Total lean mass, kg	44.5 (6.0)	63.0 (7.7)	48.6 (9.9)
Lean mass, % of body weight	54.3 (7.2)	61.5 (7.6)	55.9 (7.8)
Total fat mass, kg	36.9 (13.5)	38.1 (15.4)	37.1 (13.9)
Fat mass, % of body weight	42.9 (7.6)	35.4 (8.0)	41.2 (8.3)
ALMI, kg/m^2^	7.69 (1.02)	9.40 (1.10)	8.06 (1.25)
LMI, kg/m^2^	16.88 (1.78)	20.13 (1.94)	17.59 (2.26)
FMI, kg/m^2^	13.99 (4.93)	12.15 (4.61)	13.58 (4.92)
ALM/body weight	0.246 (0.031)	0.286 (0.032)	0.254 (0.035)

Data are presented as mean (SD), median [interquartile range], or n (%), as appropriate. Each participant contributed a single assessment, defined as the latest eligible assessment performed more than 3 years after surgery. Fifty participants also contributed to the longitudinal cohort through distinct assessments performed within the first 3 postoperative years; no DXA assessment was shared between the two analyses. ALM, appendicular lean mass; ALMI, appendicular lean mass index; BMI, body mass index; CRP, C-reactive protein; DXA, dual-energy X-ray absorptiometry; eGFR, estimated glomerular filtration rate; FMI, fat mass index; IQR, interquartile range; LMI, lean mass index; SD, standard deviation.

**Table 5:** **Association between serum prealbumin and body composition more than 3 years after Roux-en-Y gastric bypass**.

Cross-Sectional Cohort, *n* = 310				
**Panel A. Association between prealbumin and body composition**				
	**Difference per 0.05 g/L lower prealbumin** (**95% CI**)	***p***	**Partial correlation** (**95% CI**)	**95% CI excludes |r| = 0.30**
**Model 1: adjusted for sex**				
Total lean mass, kg	+0.67 (−0.19 to +1.53)	0.124	−0.088 (−0.197 to +0.024)	Yes
Lean mass, % of body weight	−0.40 (−1.38 to +0.58)	0.423	+0.046 (−0.066 to +0.156)	Yes
ALMI, kg/m^2^	+0.13 (−0.01 to +0.27)	0.067	−0.105 (−0.214 to +0.007)	Yes
LMI, kg/m^2^	+0.21 (−0.04 to +0.45)	0.096	−0.095 (−0.204 to +0.017)	Yes
ALM/body weight	−0.0008 (−0.0050 to +0.0033)	0.692	+0.023 (−0.089 to +0.134)	Yes
FMI, kg/m^2^	+0.45 (−0.20 to +1.10)	0.178	−0.077 (−0.187 to +0.035)	Yes
**Model 2: additionally adjusted for age and time since surgery**				
Total lean mass, kg	+0.64 (−0.20 to +1.47)	0.133	−0.086 (−0.196 to +0.026)	Yes
Lean mass, % of body weight	−0.09 (−1.03 to +0.85)	0.850	+0.011 (−0.101 to +0.123)	Yes
ALMI, kg/m^2^	+0.12 (−0.01 to +0.26)	0.079	−0.100 (−0.210 to +0.012)	Yes
LMI, kg/m^2^	+0.18 (−0.06 to +0.43)	0.131	−0.086 (−0.196 to +0.026)	Yes
ALM/body weight	+0.0007 (−0.0032 to +0.0046)	0.729	−0.020 (−0.131 to +0.092)	Yes
FMI, kg/m^2^	+0.23 (−0.39 to +0.86)	0.464	−0.042 (−0.153 to +0.070)	Yes
**Panel B. Association with ALMI according to time since surgery**				
	**Difference per 0.05 g/L lower prealbumin** (**95% CI**)	***p***	**Partial correlation** (**95% CI**)	
3 to <5 years (*n* = 59)	+0.21 (−0.08 to +0.49)	0.149	−0.193 (−0.432 to +0.071)	No
5 to <10 years (*n* = 119)	+0.10 (−0.11 to +0.30)	0.353	−0.087 (−0.264 to +0.096)	Yes
≥10 years (*n* = 132)	+0.10 (−0.14 to +0.33)	0.411	−0.073 (−0.242 to +0.101)	Yes
**Panel C. Body composition according to the clinical prealbumin threshold**				
	**Prealbumin < 0.20 g/L, *n* = 80**	**Prealbumin ≥ 0.20 g/L, *n* = 230**	**Adjusted difference** (**95% CI**)	***p***
Total lean mass, kg	46.42 (8.53)	49.31 (10.31)	+0.51 (−1.10 to +2.11)	0.533
Lean mass, % of body weight	55.03 (7.47)	56.17 (7.97)	+0.60 (−1.21 to +2.41)	0.515
ALMI, kg/m^2^	7.88 (1.13)	8.12 (1.29)	+0.08 (−0.19 to +0.34)	0.572
LMI, kg/m^2^	17.27 (1.97)	17.70 (2.34)	+0.14 (−0.32 to +0.61)	0.543
ALM/body weight	0.2499 (0.0327)	0.2561 (0.0358)	+0.0035 (−0.0040 to +0.0110)	0.359
FMI, kg/m^2^	13.84 (4.84)	13.49 (4.95)	−0.27 (−1.48 to +0.93)	0.658

Panel A shows associations between serum prealbumin and body-composition outcomes. Model 1 is adjusted for sex, and Model 2 is additionally adjusted for age and time since surgery. Regression coefficients represent the expected difference in each outcome per 0.05 g/L lower serum prealbumin concentration, and partial correlations are adjusted for the same covariates. “95% CI excludes |r| = 0.30” indicates whether the confidence interval of the partial correlation lies entirely between −0.30 and +0.30, thereby excluding correlations of moderate or greater magnitude. Panel B shows the association between serum prealbumin and ALMI according to time since surgery (3 to <5 years, 5 to <10 years, and ≥10 years), with adjustment for sex and age. Panel C compares body-composition outcomes between participants with serum prealbumin <0.20 and ≥0.20 g/L. Values are presented as mean (SD), and adjusted differences were estimated using linear regression controlling for sex, age, and time since surgery. ALM, appendicular lean mass; ALMI, appendicular lean mass index; CI, confidence interval; FMI, fat mass index; LMI, lean mass index; SD, standard deviation.

## Data Availability

The data supporting the findings of this study are available from the corresponding author upon request.

## Supplementary Materials

The following supporting information can be downloaded at: https://www.mdpi.com/article/10.3390/nu18213580/s1, Figure S1: Discrimination of serum prealbumin across different thresholds of postoperative lean mass loss; Table S1: Preoperative characteristics of patients with and without an available 3-year assessment.

## Abbreviations

The following abbreviations are used in this manuscript:

ALM	Appendicular lean mass
ALMI	Appendicular lean mass index
DXA	Dual-energy X-ray absorptiometry
LM	Lean mass
LMI	Lean mass index
MBS	Metabolic bariatric surgery
RYGB	Roux-en-Y gastric bypass
TWL	Total weight loss
