# Supplementary material for: Limited Clinical Utility of Serum Prealbumin for Assessing DXA-Derived Lean Mass After Roux-en-Y Gastric Bypass: Longitudinal and Long-Term Cross-Sectional Analyses
Source: Nutrients. 2026 Oct 29;18(21):3580. doi: 10.3390/nu18213580 (PMC13764954; doi:10.3390/nu18213580)
Supplement: Supplementary file 1 [file nutrients-18-03580-s001.zip › nutrients-4605296-supplementary.pdf]

Table S1. Preoperative characteristics of patients with and without an available 3-year assessment.

	With 3-year DXA	Without 3-year DXA	*P*	SMD
**n**	**77**	**101**	—	—
Women, n (%)	55 (71)	69 (68)	0.78	—
Age, years	44.0 (11.3)	43.0 (10.8)	0.55	+0.09
Height, m	1.67 (0.09)	1.67 (0.10)	0.89	−0.02
Body weight, kg	116.1 (21.3)	122.0 (25.4)	0.10	−0.25
BMI, kg/m^2	41.6 (5.4)	43.5 (6.2)	0.03	−0.33
Biochemistry				
Prealbumin, g/L	0.264 (0.042)	0.256 (0.038)	0.24	+0.18
Prealbumin < 0.20 g/L, n (%)	1 (1)	4 (4)	0.39	—
CRP, mg/L	3.8 [2.5–6.0]	4.1 [2.3–7.0]	0.58	−0.11
Body composition (DXA)				
Total lean mass, kg	54.8 (10.8)	57.7 (12.4)	0.10	−0.25
Lean mass, % of body weight	47.6 (5.3)	47.9 (5.6)	0.73	−0.05
Total fat mass, kg	57.7 (13.3)	60.4 (15.5)	0.22	−0.18
Fat mass, % of body weight	49.9 (5.6)	49.7 (5.8)	0.76	+0.05
ALMI, kg/m^2	9.53 (1.29)	9.92 (1.44)	0.06	−0.29
LMI, kg/m^2	19.56 (2.27)	20.52 (2.64)	0.01	−0.39
FMI, kg/m^2	20.80 (4.35)	21.63 (4.80)	0.23	−0.18
ALM / body weight	0.230 (0.029)	0.229 (0.029)	0.88	+0.02

Supplementary Table S1. Preoperative characteristics of participants with and without an available 3-year DXA assessment in the longitudinal cohort. The group without 3-year assessment included 69 participants who had not yet reached this time point and 32 with missing assessments. Data are presented as mean (SD), median [interquartile range] or n (%), as appropriate.

P values are from Welch two-sample t-tests for continuous variables, a Wilcoxon rank-sum test for C-reactive protein, a continuity-corrected two-sample test of proportions for sex, and Fisher's exact test for prealbumin <0.20 g/L. SMDs are signed as the group with a 3-year assessment minus the group without one; categorical SMDs are not reported.

ALM, appendicular lean mass; ALMI, appendicular lean mass index; BMI, body mass index; CRP, C-reactive protein; DXA, dual-energy X-ray absorptiometry; FMI, fat mass index; IQR, interquartile range; LMI, lean mass index; SD, standard deviation; SMD, standardized mean difference.

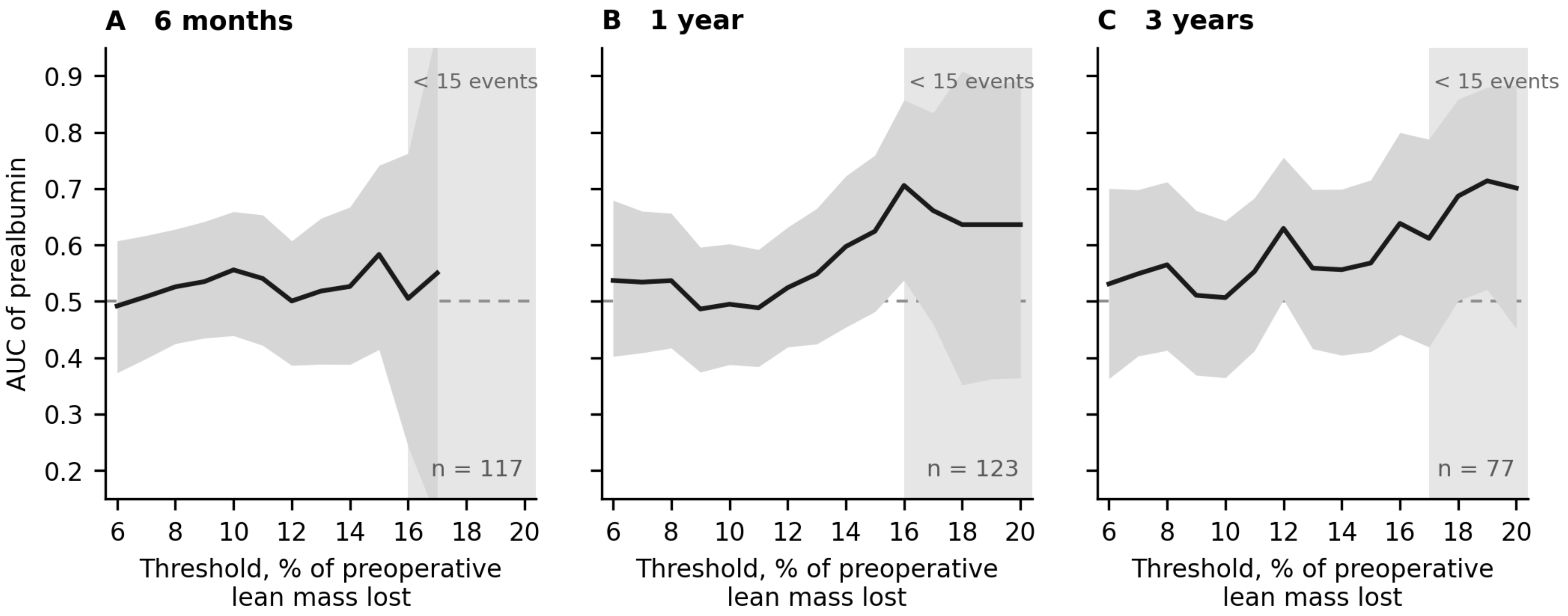

Figure S1. Discrimination of serum prealbumin across different thresholds of postoperative lean mass loss.
At each threshold on the horizontal axis, the target condition was defined as loss of more than the specified percentage of preoperative lean mass. Receiver operating characteristic analyses were performed separately at 6 months (A), 1 year (B), and 3 years (C), with prealbumin oriented a priori so that lower concentrations represented greater predicted risk. Solid lines show the area under the receiver operating characteristic curve (AUC), shaded bands the 95% confidence intervals, and the dashed horizontal line no discrimination (AUC = 0.50). Shaded vertical regions indicate thresholds yielding fewer than 15 cases; AUCs were not estimated when either outcome group contained five or fewer participants.
AUC, area under the receiver operating characteristic curve; CI, confidence interval
